# Alternating Gyroid Network Structure in an ABC Miktoarm Terpolymer Comprised of Polystyrene and Two Polydienes

**DOI:** 10.3390/nano10081497

**Published:** 2020-07-30

**Authors:** Dimitrios Moschovas, Gkreti-Maria Manesi, Andreas Karydis-Messinis, George Zapsas, Konstantinos Ntetsikas, Nikolaos E. Zafeiropoulos, Alexey A. Piryazev, Edwin L. Thomas, Nikos Hadjichristidis, Dimitri A. Ivanov, Apostolos Avgeropoulos

**Affiliations:** 1 Department of Materials Science Engineering, University of Ioannina, University Campus-Dourouti, 45110 Ioannina, Greece; dmoschov@cc.uoi.gr (D.M.); gretimanesi@cc.uoi.gr (G.-M.M.); karydis.and@gmail.com (A.K.-M.); nzafirop@uoi.gr (N.E.Z.); 2 Faculty of Chemistry, Lomonosov Moscow State University (MSU), GSP-1, 1-3 Leninskiye Gory, 119991 Moscow, Russia; dimitri.ivanov@uha.fr; 3 Physical Sciences and Engineering Division, KAUST Catalysis Center, Polymer Synthesis Laboratory, King Abdullah University of Science and Technology (KAUST), Thuwal 23955, Saudi Arabia; georgios.zapsas@kaust.edu.sa (G.Z.); konstantinos.ntetsikas@kaust.edu.sa (K.N.); nikolaos.hadjichristidis@kaust.edu.sa (N.H.); 4 Institute of Problems of Chemical Physics, Russian Academy of Sciences, Chernogolovka, 142432 Moscow, Russia; stunnn@gmail.com; 5 Institut de Sciences des Matériaux de Mulhouse—IS2M, CNRS UMR7361, 15 Jean Starcky, 68057 Mulhouse, France; 6 Department of Materials Science and NanoEngineering, Rice University, Houston, TX 77030, USA; elt@rice.edu

**Keywords:** miktoarm star terpolymer, anionic polymerization, chlorosilane chemistry, characterization in solution, structural characterization, DSC, TEM, SAXS, tricontinuous network

## Abstract

The synthesis, molecular and morphological characterization of a 3-miktoarm star terpolymer of polystyrene (PS, ${\bar{M}}_{n}$ = 61.0 kg/mol), polybutadiene (PB, ${\bar{M}}_{n}$ = 38.2 kg/mol) and polyisoprene (PI, ${\bar{M}}_{n}$ = 29.2 kg/mol), corresponding to volume fractions (φ) of 0.46, 0.31 and 0.23 respectively, was studied. The major difference of the present material from previous ABC miktoarm stars (which is a star architecture bearing three different segments, all connected to a single junction point) with the same block components is the high 3,4-microstructure (55%) of the PI chains. The interaction parameter and the degree of polymerization of the two polydienes is sufficiently positive to create a three-phase microdomain structure as evidenced by differential scanning calorimetry and transmission electron microscopy (TEM). These results in combination with small-angle X-ray scattering (SAXS) and birefringence experiments suggest a cubic tricontinuous network structure, based on the I4_1_32 space group never reported previously for such an architecture.

## 1. Introduction

ABC linear triblockterpolymers consist of two-junction points connecting the two end-blocks (A and C) to the center block (B), whereas for ABC miktoarm stars a single central junction point joins the three different arms [1,2,3,4,5,6,7,8,9,10,11,12]. The morphology of ABC terpolymers is also influenced by two independent composition variables (ф_A_, ф_B_, ф_C_ = 1 − ф_A_ − ф_B_) and by three interaction parameters (*χ*_AB_, *χ*_BC_, and *χ*_AC_). For linear terpolymers, the block sequence can affect the microphase morphology [1,7,8,9], whereas, in the case of miktoarm stars, the nonlinear architecture excludestheblock sequence as a depending factor on the finally adopted morphology. When the blocks are selected to exhibit relatively high values *χ*_AB_N_AB_, *χ*_BC_N_BC_, and *χ*_AC_N_AC_ for all three possible pairs (AB, BC and AC respectively), a three-microphase structure will arise [13].

Experimental and theoretical studies on linear ABC terpolymer microstructures in bulk demonstrated many unique microdomain morphologies as reviewed by Hadjichristidis and coworkers more than a decade ago [13]. Continuous network morphologies of two or more chemically different phases are of main interest due to their enhanced mechanical and transport properties as well as owing to their potential use in technologies such as catalysis, photonic materials, solar cells, and separation techniques through improved membranes. Based on potential technological applications for such materials, an additional review has been published by Meuler et al. for ABC linear terpolymers and the adopted structure/properties relationship is thoroughly described [14]. 

It should be pointed out that the one junction point restriction for star ABC type terpolymershas forced the finally adopted morphology to be explained by considering that the three different phases lie on periodically spaced parallel lines defined by the necessity of mutual intersection of the three different blocks. This behavior and the finally observed morphology on nonlinear ABC terpolymers is completely different compared to linear block copolymers and terpolymers [15].

The first ABC miktoarm star terpolymer reported in the literature [16] was comprised of polystyrene (PS), 1,4-PB and 1,4-PI. This material exhibited only two distinct microdomain regions [17], a set of hexagonally close packed cylindrical PS domains in a mixed polydiene matrix due to the low interaction parameter between polybutadiene (PB) and polyisoprene (PI). Moreover, the miktoarm architecture affected the microphase separated structure, leading to hexagonally packed cylinders instead of a lamellar or a bicontinuous network structure, as expected for the corresponding non linear copolymers of the A_2_B copolymers with an equivalent volume fraction of PS, predicted already theoretically by Milner [18]. 

The morphological characterization of a three-phase microdomain structure can be very challenging since different staining protocols may be needed for TEM investigation. Moreover, in SAXS experiments, the scattering depends on the three pairwise electron density differences: (ρ_1_ − ρ_2_)^2^, (ρ_1_ − ρ_3_)^2^, (ρ_2_ − ρ_3_)^2^, as well as on the microdomain geometry. Ruthenium tetroxide (RuO_4_) is preferred for staining of the PS chains and osmium tetroxide (OsO_4_) for staining of the corresponding polydiene blocks. Initially, it was believed that in general the various polydiene blocks would stain approximately identically. However, it seems that OsO_4_ staining depends on the geometric isomerism (-1,4, -1,2, -3,4) of the most studied polydienes (PB and PI). It has already been reported in the literature [19] through TEM studies that the PI block with high 3,4 content (~55–60%) is stained less with OsO_4_ than the high 1,4-PB component (~90–92% -1,4). In the specific study [19] a detailed analysis of time depending staining procedure with OsO_4_ for the two different polydienes is also reported.

The synthesis of miktoarm star terpolymers consisting of three chemically different components has been reported several times in the literature [13]. More recently, Ito et al. have reviewed the synthesis of miktoarm star polymers (among them ABC non linear architectures) based on anionic polymerization methods focusing exclusively on well-defined final polymers with precisely controlled architectures [20].

Fujimoto et al. [21] synthesized stars of PS, poly(dimethylsiloxane) (PDMS) and poly(tert-butylmethacrylate) (PtBMA); Huckstadt et al. prepared three-miktoarm stars of PS, 1,2-PB and poly(2-vinylpyridine) (P2VP) [22] or poly(methylmethacrylate) (PMMA) [23]; Sioula et al. [24,25] prepared stars of PS, 1,4-PI and PMMA; Zioga et al. [26] prepared stars of PS, 1,4-PI and poly(2-vinylpyridine) (P2VP); Bellas et al. [27] prepared stars of PS, 1,4-PI and PDMS; Lambert et al. [28] synthesized miktoarm terpolymers of PS, poly(ethylene oxide) (PEO) with PMMA or poly(*ε*-caprolactone) (PCL) or poly(L-lactide) (PLLA), whereas Nasser-Eddine et al. [29] prepared corresponding materials with PS, PtBMA and PEO segments respectively. By incorporating specific 1,1-diphenylethylene derivatives as coupling agents Hirao et al. [30] have prepared a vast number of non linear three-component polymer systems such as(PB)(PS)(P2VP), (PDMS)(PS)(PtBMA) and (PS)(PMMA)(PEO). Matsushita et al. [31] prepared three-miktoarm star terpolymers of the (PI)(PS)P2VP) type using a different synthetic procedure than the one followed by Zioga et al. [25] Furthermore, Liu et al. have demonstrated the facile synthesis of ABC star terpolymers consisting of PS, poly(*ε*-caprolactone) and poly(phenyl isocyanide) blocks through living polymerization methods [32].

Below we review the microdomain structures of nine (9) three-arm stars comprised of three different components.

The morphology of the (PDMS)(PS)(PtBMA) star polymers prepared by Fujimoto et al. [21], was investigated by Okamoto et al. [33] using DSC (differential scanning calorimetry), TEM and SAXS. From TEM experiments, the PDMS domains exhibited three-fold symmetry but the remaining two microphases were not distinguishable. Ordered tricontinuous structures were observed, which was a behavior completely different from what should have been expected, based on the initial volume fraction ratios of the different components.

The (PS)(PI)(PMMA) stars synthesized by Sioula et al. [34,35], led to new interesting structures and, depending on the composition of the three components, either two domain structures were observed [34] or the junction points were confined onto lines where all three different microphases intersected [35]. The first case was adopted due to partial miscibility between PS and PMMA arms, leading to new 2D periodic structures where the junction points were located on the PS/PI Intermaterial Dividing Surface (IMDS). When the total volume fraction of PS and PI was ~0.40 the microdomain structure consisted of constant mean curvature (CMC) coaxial cylinders (plane group symmetry p6mm) on a 2D hexagonal lattice.In the case of total volume fraction of PS and PI of 0.56 and 0.78, respectively, a novel two-dimensionally periodic non-CMC concentric rhombohedral morphology (plane group symmetry p2mm) is observed which is the only diamond prism-shaped IMDS in block copolymers reported in the literature. 

For stars examined by Lambert et al. [28] containing two crystallizable blocks [(PS)(PEO)(PCL)], the crystallization started from the homogeneous melt phase and concluded to microphase separation [36]. Different superstructures were formed (spherulites/axialites) depending on the type of the crystallizable block (PEO and/or PCL), indicating a pure PEO crystalline component of the amorphous blocks with the PCL crystal.

The morphologies found by Abetz’s group [22] for the (PS)(PB)(P2VP)-type miktoarm stars include dense packing of cylinders with tetragonal or hexagonal order, when the systems exhibited lower volume fractions of P2VP, indicating that the junction points between the three different segments lie along lines, as already mentioned by Sioula et al. [35].

Finally, the microstructure observed by Zioga et al. [26] exhibited two kinds of hexagonally packed adjoined cylinders (PI and P2VP) in a PS matrix, forming regular curved hexagons, by using preferential stains (either OsO_4_ or CH_3_I for the PI or the P2VP phase respectively), where, again, the junction points are confined on parallel and periodically spaced lines which are comprised by the intersection of the three microdomain interfaces.

Matsushita et al. [37] studied a large number of miktoarm star terpolymers of the (PI)(PS)(P2VP) type and terpolymer/homopolymer blends, which indicated various periodic tiling patterns consisting of polygons, core-shell-type topologies, three-phase component structures, cubic networks, etc., trying to cover a large range of possible volume fraction combinations. Within their extensive study, they have assigned one of the observed microstructures of a (PI)(PS)(P2VP) terpolymer blended with PS homopolymer in the zinc blende type structure with a 45 mm lattice constant, which is a non closed packed structure impossible to be achieved by using linear triblock terpolymers [38].

It is obvious from the above that no-one up to date has observed a three-dimensional cubic structure in such complex architectureterpolymers, probably due to symmetry or asymmetry constrains as well as due to the use of blocks which varied significantly in chemical structure. 

The most frequently encountered ordered bicontinuous network structure in block copolymers (BCPs) is the Double Gyroid (DG). This structure exhibits two 3D continuous, interpenetrating but nonintersecting enantiomeric networks of the minority component (usually 32–36 vol% for diblock copolymers) in the majority matrix. The networks exhibit nodes with three-fold symmetry. (The Ordered Bicontinuous Double Diamond (OBDD) structure was originally reported for linear diblock copolymers, but samples initially believed to exhibit such a structure were revaluated [39] and found to exhibit DG). Only one sample with an inverse star copolymer architecture and asymmetry factor between outer and inner blocks equal to 4 exhibits the OBDD morphology [40], where the two networks are identical and have nodes with tetrahedral symmetry. Bates’ group [41,42,43] has studied a large number of ABC linear triblock terpolymers consisting of PI, PS and PEO. Multiple continuous network morphologies were discovered for certain volume fractions of PEO (0.1 ≤ *φ*_ΡΕO_ ≤ 0.3). The detailed structures of these periodic network morphologies were determined via TEM, SAXS, DSC, DMS (dynamic mechanical spectroscopy) and birefringence experiments. The core-shell double gyroid (Ia$\bar{3}$d space group), a gyroid with two different component networks (I4_1_32 space group) [42] and a novel orthorhombic network phase (Fddd space group) were observed. The orthorhombic network phase was the structure initially obtained as reported in the literature [41].

More complex architectures have been reported in the literature by the Avgeropoulos’ group [44] involving synthesis approaches through combinations of anionic and atom transfer radical polymerization methods resulting to well-defined star triblock terpolymers of the (PS-*b*-P2VP-*b*-PEO)_3_ type leading to unique results on their properties in solution and in bulk.

Moreover, complex architectures of second-generation dendritic copolymers consisting of polymeric chains of 1,4-PB and high 3,4-PI (approximately 54% 3-4-microstructure)—[(PB)_2_PI]_3_ exhibited hexagonally packed cylinders of the 3,4-PI in the matrix of PB [45]. Second-generation dendritic terpolymers consisting of PS, 1,4-PB and high 3,4-PI demonstrating the same 3,4-PI content have been also synthesized [46] in order to increase our knowledge towards the effect of block connectivity in the aforementioned system. Well-organized three-phase microstructures were also observed [47].

Furthermore, it should be mentioned that in such ABC linear terpolymers of the PS-*b*-PB_1,4_-*b*-PI_3,4_ sequence our research group has managed [48] to identify the core-shell DG morphology concluding to the fact that the PI segment with the specific percentage of 3,4-microstructure is actually leading to a relatively high Flory–Huggins interaction parameter not only with PS but also with PB_1,4_. We have also demonstrated the stability of the three-phase four-layer lamellar structure by synthesizing heptablock quaterpolymers of the sequence PS-*b*-PB_1,4_-*b*-PI_3,4_-*b*-PDMS-*b*-PI_3,4_-*b*-PB_1,4_-*b*-PS [49]. The results from this specific work [49] indicated that the PI_3,4_ and PDMS blocks are miscible, verifying our assumption of high value for the Flory–Huggins interaction parameter between the specific PI and PS or PB_1,4_.

Very recently the Hadjichristidis’ group [50] have reported the synthesis and structural characterization of polyethylene (PE) based star terpolymers of various types for the first time where the PE block has not been prepared by hydrogenation of the usual PB block synthesized by anionic polymerization exhibiting approximately 92% -1,4 and 8% -1,2 microstructures. The synthesis procedure involved a combination of anionic polymerization, polyhomologation and linking reactions. The same group has been pursuing star terpolymers of various types involving ABC nonlinear architectures as evident by recent publications without observing three-dimensional cubic structure in such complex architecture terpolymers [51,52].

In this paper, we report the synthesis and the structural characterization of a new three-miktoarm star terpolymer consisting of PS, 1,4-PB and high 3,4-PI (approximately 54% 3-4-microstructure from ^1^H-NMR). This work builds on our previous observations [19] where a three-phase four-layer lamellar structure and a new cylindrical morphology occurred in linear triblockterpolymers of the PB-*b*-PS-*b*-PI and the PS-*b*-PI-*b*-PB type having the same 3,4 content in the PI block. The star terpolymer synthesized in this work exhibited low dispersity index (*Đ* = 1.07) and the respective ratio of PS/PB/PI volume fractions was approximately equal to 2/1/1 (since the volume fractions of PS, PB and PI were equal to 0.46, 0.31 and 0.23 respectively. 

## 2. Materials and Methods 

The successful synthesis of the 3-miktoarm star terpolymer was accomplished by anionic polymerization. The specific polymerization method involves the use of the high-vacuum technique in evacuated, n-BuLi-washed, benzene-rinsed glass vessels. All purification procedures for monomers (styrene, butadiene and isoprene), solvents (benzene, THF) and initiator (use of commercially available sec-BuLi diluted in heptanes and further dilution until desired concentration) to the standards required for anionic polymerization have been described in detail elsewhere (all reagents were purchased from Sigma-Aldrich Co., St Louis, MO, USA) [47,53].

The molecular characterization was carried out via size exclusion chromatography (SEC), low-angle laser light scattering (LALLS) and membrane osmometry (MO). For the SEC experiments with THF as eluent operated at 30 °C, a PL-GPC 50 Integrated GPC System from Agilent Technologies (St. Clara, CA, USA) was employed. For the LALLS measurements (for solutions in THF at 25 °C), a Milton Roy Chromatix KMX-6 (Philadelphia, PA, USA) low-angle laser photometer equipped with a helium-neon laser and operated at a wave-length of 633 nm was used. The MO measurements were carried out at 35 °C using dried toluene as solvent in a Gonotec 090 (Berlin, Germany) membrane osmometer. More details are given elsewhere [19].

Proton nuclear magnetic resonance (^1^H-NMR) spectroscopy was used for the determination of the composition and the microstructure of the different segments. The experiments were carried out in CDCl_3_ at 30 using a Varian Unity Plus 300/54 instrument. For the PB block, the typical microstructure observed was identical to what is expected when anionic polymerization of butadiene in benzene (approximately 92 wt % -1,4 and 8 wt % -1,2) is performed. For the PI block, the observed microstructure was different than that of the usual anionic polymerization of isoprene in benzene. Actually, the small quantity of polar solvent used (THF, <1mL) leads to a microstructure of PI of 54 wt % -3,4, 16 wt % -1,2 and 30 wt % -1,4. The lower 1,4 content is due to the presence of the polar solvent. For this study the small amount of THF is added only to obtain the desired microstructures of the PI chains, in order to compare the linear and the star samples, since each chain is synthesized independently and then added to the methyltrichlorosilane, used for the linking reaction. 

Differential scanning calorimetry (DSC) experiments, employing a TA Instruments 2910 Modulated DSC (TA Instruments Ltd., Leatherhead, England), were conclusive for the microphase separation between the three different segments. Different glass transition temperatures were observed at values of: T_g1_ ~ −80 °C, T_g2_ ~ −30 °C and T_g3_ ~ 100 °C. These values are considered to be very close to those already reported by Avgeropoulos et al. [19]. The lowest and highest values correspond approximately to 1,4-PB and PS respectively [54]. PI homopolymers [55] with increased 3,4-microstructure have been examined with DSC and the T_g_varied from−25 °C to −30 °C.

Transmission electron microscopy (TEM) and small-angle X-ray scattering (SAXS) were employed for morphological characterization of the terpolymer. More details are given elsewhere [56]. Approximately 0.7 mm-thick films of the material were cast from a dilute solution (~4 wt %) in toluene, over a period of a week at ambient conditions. The selectivity of the solvent can be estimated by examination of the solubility parameters, δ [57]. The literature values of δ for polystyrene and 1,4-polybutadiene (92 wt%) are 9.1 and 8.4 (cal/cm^3^)^1/2^, respectively, and that of toluene is 8.9 (cal/cm^3^)^1/2^. The high 3,4-microstructure of PI suggests a solubility parameter larger than that for cis-1,4-PI (90 wt %) [δ = 8.2 (cal/cm^3^)^1/2^]. From the above, the two polydienes should drive the disorder to order transition. Additionally, the solvent from which the films were cast is a nonselective solvent for all three components. Based on the solubility parameters, the two dienes are less soluble in toluene than the PS segments; therefore toluene is more preferential for the PS blocks. 

In order to obtain near-equilibrium microstructures after casting, the films were annealed for seven days at 130 °C under vacuum. For TEM investigation, 500–1500 Å thick sections were cryomicrotomed at −90 °C in a cryo-ultramicrotome Leica EM UC7 from Leica Microsystems (Wetzlar, Germany) and the sections were picked up on 600-mesh copper grids (Electron Microscopy Sciences, Hatfield, PA, USA). The grids were then placed in the vapors of a OsO_4_ 4% aqueous solution (Science Services, Munich, Germany) for selective staining of the two polydiene domains. The staining process was time-dependent and better contrast between the two polydienes was evident from the TEM studies after staining for approximately 30 min. A JEOL 200CX electron microscope (JEOL Ltd., Tokyo, Japan), operated at 200 kV in the bright field mode, was used to examine the stained sections.

The SAXS (diffraction) patterns were obtained at room temperature on the Time-Resolved Diffraction Facility (station X12B) at the National Synchrotron Light Source at Brookhaven National Laboratory (BNL) using a custom-built two-dimensional detector (10 × 10 cm, 512 × 512 pixels) interfaced to a real-time histogramming memory system [58]. The mass densities of PI and PB are nearly equivalent (0.903 and 0.9 g/mol, respectively) compared to that of the PS (1.06 g/mol). Since the electron densities of the two polydiene chains are approximately equal, the SAXS patterns reflect to scattering from a two-density system (pseudo-two-phase system instead of the three-phase system clearly identified by TEM). It should be noted that in the case of the PI the density of PI with ~92% -1,4 microstructure is being used, since the density of the PI with increased -3,4 microstructure has not yet been reported.

## 3. Results and Discussion

**Scheme 1:**
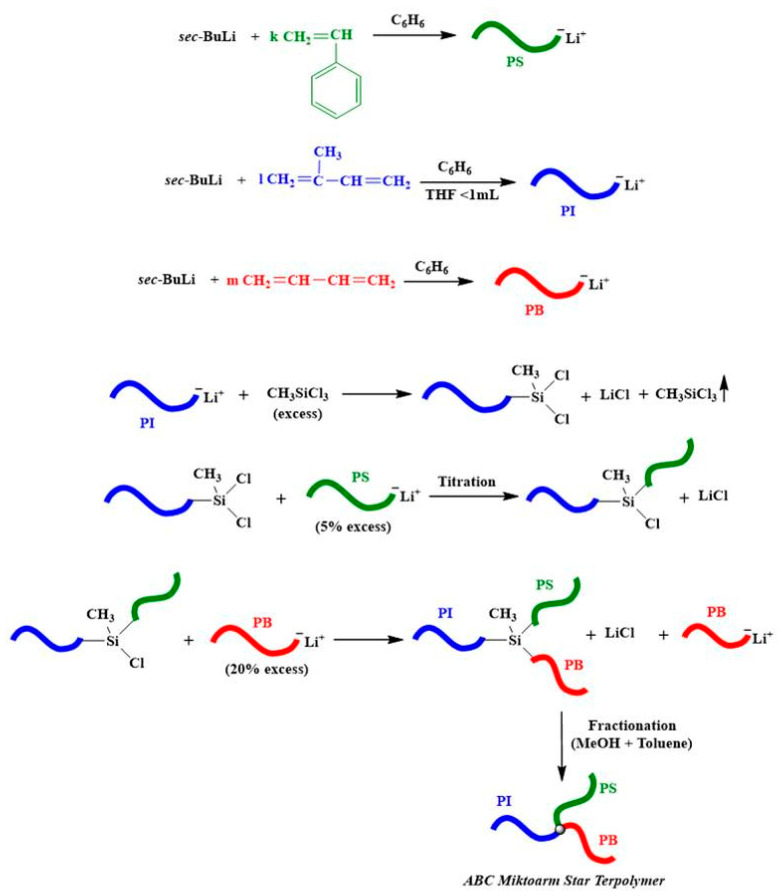
Reactions scheme for the synthesis of the final 3-miktoarm star terpolymer.

**Synthesis.** The ABC miktoarm star terpolymer was synthesized by adopting the known chlorosilane chemistry approach. In the case of linear terpolymers sequential addition of monomers is employed, whereas in the case of the miktoarm star terpolymer sequential addition of the living chains is necessary. It should be noted that the sequence of the chains added is crucial. The procedure is shown in Scheme 1.

It is evident from the reactions in Scheme 1 that each arm (PS, PI and PB) is synthesized separately and precautions are taken (addition of polar solvent) during the PI-arm formation in order for this block to exhibit increased 3,4-microstructure (~54% wt from ^1^H-NMR spectroscopy experiments). The initial step of the synthesis involved the reaction of first the PI with a very large excess of (CH_3_)SiCl_3_ (at least 300:1 for [Si–Cl]/[living ends]). 

**Figure 1:**
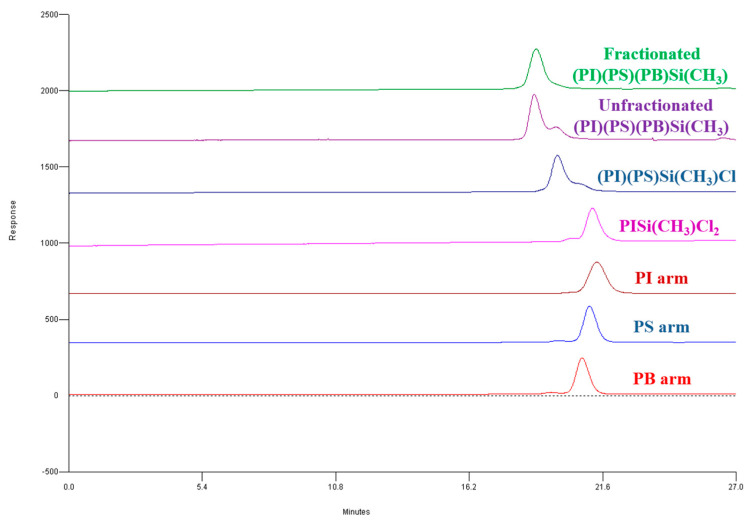
SEC chromatographs of the precursors (PS), (PI-3,4, 55%), (PB), the intermediate products and the final fractionated 3-miktoarm star terpolymer (PS)(PI)(PB)SiCH_3_.

The remaining linking reagent together with the solvents (benzene/THF) was removed under vacuum line conditions. The synthesis of the star terpolymer is based on: (a) the inability of the sterically hindered, styryl anion to completely substitute the remaining chlorine atoms of the linking reagent and (b) the ability of the less hindered butadienyl anion to react completely with the monofunctional macromolecular linking agent. Such observations indicate that the sequence of the added block is very critical. During the titration reaction, aliquots of the solution were taken in order to observe the process of the reaction via SEC. More details concerning the synthesis procedures of such nonlinear materials are given elsewhere [16]. The chromatographs from SEC for the initial blocks, the intermediate products and the final material are exhibited in Figure 1. 

The good agreement between the molecular characterization results and those calculated initially, as reported in Table 1, indicates that the final miktoarm star terpolymer can be considered a model polymer.

**Estimation of the Flory–Huggins interaction parameter (*χ*).** The fact that the two polydienes reported here are immiscible can be attributed to the positive value of *χ* and their quite large molecular weights. 

The *χ_ij_* parameter can be estimated from the solubility parameters of a pair of polymer chains *i* and *j* as follows:

(1) $$\chi_{ij}=\frac{V}{k_{B}T}{\left(\delta_{i}-\delta_{j}\right)}^{2}$$

where, *χ_ij_* is the Flory–Huggins interaction parameter between segments *i* and *j*, *k_B_* is the Boltzmann constant, *δ_i_* and *δ_j_* are the solubility parameters of chains i and j respectively, *T* (°K) is the annealing temperature of preparation of the sample for TEM and SAXS and V is the geometric average of the molecular volume of the monomeric unit of each type i. The resulting *χ_ij_* for the different segments *i* and *j* as calculated from Equation (1) are exhibited in Table 2. In Table 2, the interaction parameter for known segment-pairs is also given. The values measured experimentally in the literature [59,60] and those estimated using Equation (1) are in rather good correspondence. 

**Structural Characterization.** The major objective for synthesizing this miktoarm star terpolymer with PI exhibiting increased 3,4 microstructure, was in part to examine whether or not the star architecture forces mixing of the two polydienes at a specific molecular weight or leads to three different types of microdomains, as already reported in the literature [19] for the corresponding linear triblockterpolymers.

From the DSC experiments, it is straightforward that the two polydienes are completely immiscible since the T_g_ values are almost identical to those obtained for the corresponding homopolymers (already mentioned above). Different glass transition temperatures were observed at values of: T_g1_ ~ −80 °C, T_g2_ ~ −30 °C and T_g3_ ~ 100 °C eventually attributed to the PB, PI and PS blocks respectively. The T_g_ of the PI segments is differentfrom the corresponding ones of high 1,4-PI (~−60 °C) and high 1,4-PB (~−80 °C), as already known by the literature [19]. DSC measurements clearly indicate three distinctive T_g_s suggesting a three-phase microdomain structure.

The structural symmetry of the polymer was initially studied through optical birefringence measurements. It is known that materials exhibiting cubic symmetry show no birefringence, since all second rank tensor properties (e.g. optical) of cubic crystals are isotropic. Noncubic structures are optically anisotropic and as a result, exhibit nonzero birefringence. The film of the terpolymer revealed approximately zero birefringence (0.003), indicating that the material is either homogeneous or cubic. 

**Figure 2:**
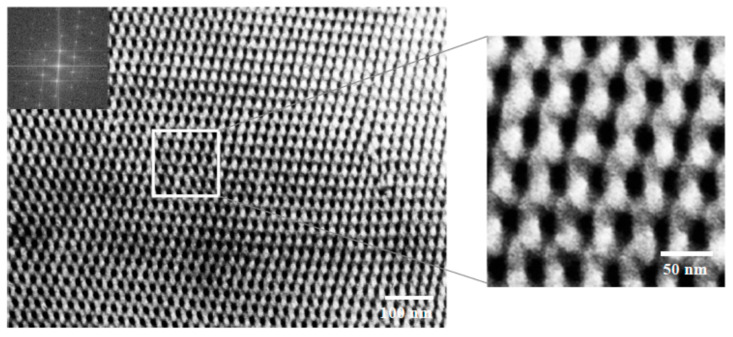
Bright-field TEM image and a “magnified area” for enhancing details, exhibiting approximately a [110] projection. The insert at the upper left of the TEM image is the corresponding FFT (fast Fourier transform). White areas correspond to PS domains, grey areas to 3,4-PI blocks and the black areas to the PB segments after staining with OsO_4_ for approximately 30 min.

**Figure 3:**
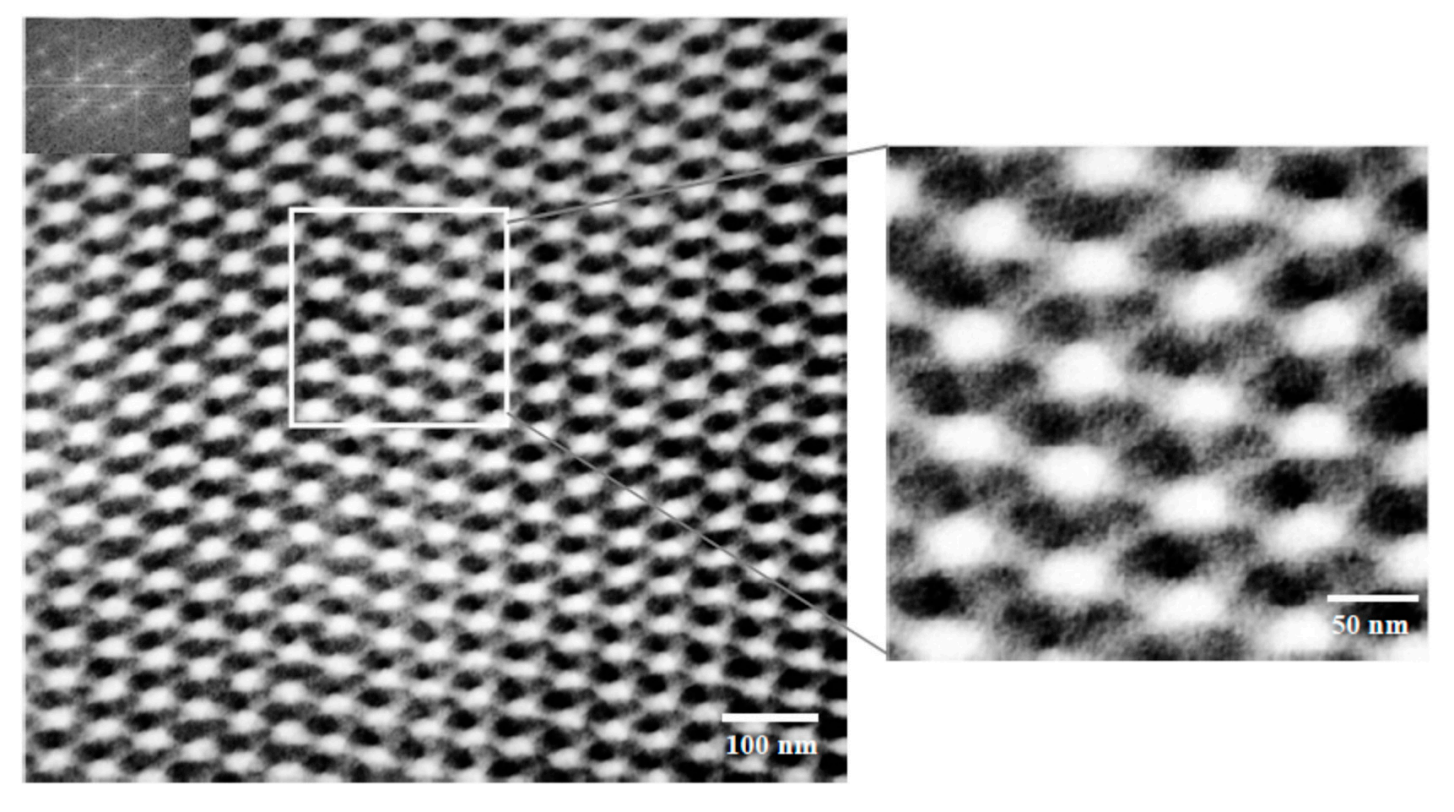
Bright-field TEM image, and a “magnified area” for enhancing details, exhibiting approximately a [111] projection. The insert at the upper left of the TEM image is the corresponding FFT. White areas correspond to PS domains, grey areas to 3,4-PI blocks and the black areas to the PB segments after staining with OsO_4_ for approximately 30 min.

**Figure 4:**
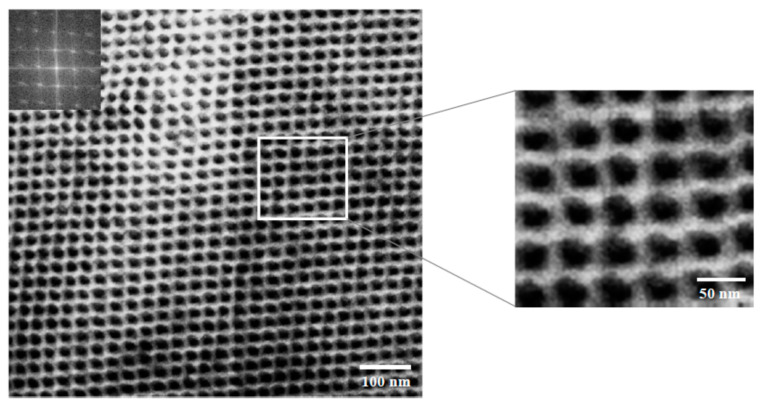
Bright-field TEM image, and a “magnified area” for enhancing details, exhibiting approximately a [100] projection. The insert at the upper left of the TEM image is the corresponding FFT. White areas correspond to PS domains, grey areas to 3,4-PI blocks and the black areas to the PB segments after staining with OsO_4_ for approximately 30 min.

**Figure 5:**
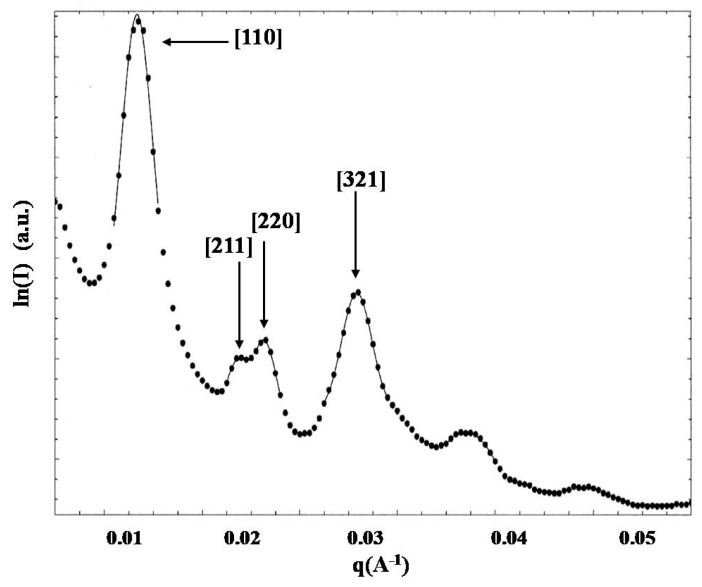
lnI(q) vs. q plot of the 3-miktoarm star terpolymer. The observation of a large number of reflections indicates a highly ordered material.

Selected bright-field TEM micrographs of the SBI star sample exhibiting grains with approximately 2-fold ([110]), 6-fold ([111]) and 4-fold ([100]) projections are given in Figure 2, Figure 3 and Figure 4 respectively. Despite the fact that the sample has a complex nonlinear architecture, excellent long-range order is observed in the TEM images (as can be seen in Figure 2, Figure 3 and Figure 4) and by the multiple Bragg peaks in the SAXS pattern (Figure 5). The approach we use in this paper is to eliminate candidate space groups by using observations from the experimental TEM images, SAXS plots and considerations given to the geometrical requirements and implications of the observed interconnected structures.

In order to identify a cubic structure, crystallographic elements should be established. The permitted reflections for each of the 17 cubic aspects are noted in the International Tables for X-Ray Crystallography [61] and could lead to the space group corresponding to our observations. The initial results from the TEM images do not correspond to those observed for the DG and OBDD cubic structures with $Ia\bar{3}d$ and $Pn\bar{3}m$ space groups, respectively [56]. Such observations, together with the SAXS data, could minimize the number of possible cubic space groups. The SAXS data is exhibited in Figure 5, where at least six distinct reflections are observed. 

The q_n_/q_1_ ratio of these peaks is: 1.0, 1.79, 1.96, 2.68, 3.50 and 5.22. These ratios do not match the two cubic structures (OBDD and DG) extensively studied already in the block copolymer literature. Actually the first four reflections are noted approximately as $\sqrt{1}$, $\sqrt{3}$, $\sqrt{4}$ and $\sqrt{7}$, which are those of a p6mm hexagonal structure, possibly misleadingly suggesting a hexagonally cylindrical morphology, but this structure is ruled out by the lack of birefringence as already discussed above. Since the hkl indices for the cubic aspects do allow a value of *s* = *h*^2^ + *k*^2^ + *l*^2^ = 7, therefore the aforementioned four reflections should be multiplied by a factor of $\sqrt{2}$, corresponding to the values of $\sqrt{2}$, $\sqrt{6}$, $\sqrt{8}$ and $\sqrt{14}$.

A thorough inspection of the international tables of crystallography [61] leads to a possible candidate space group: I4_1_32 where the first three permitted reflections are: [110], [211] and [220]. The permitted reflections from the I4_1_32 space group and those observed experimentally are given in Table 3. No other cubic space group gives this set of allowed reflections, hence setting: q2q1=62=31, as can be clearly understood in Table 3, from the comparison with the permitted reflections of OBDD and DG (these two cubic space groups are compared with I4_1_32, since they are the only cubic space groups observed in polymeric samples).

It should be noted that, if this space group is employed, then the fourth reflection should be assigned as [321]. However, the two following ones are missing ([310] and [222], respectively) despite the long-range order exhibited in the TEM images. The SAXS pattern does not indicate any amalgamation of the missing peaks, leading to the possibility of extinction due to a minimum in the form factor depending on the volume fractions of each block. Actually, careful consideration of the SAXS pattern implies that the first four peaks are rather sharp, when the remaining three indicate a possible overlap of two, three or even more reflections, since they lack sharpness.

The major finding in this paper is the fact that the cubic structure discovered for the synthesized 3-miktoarm star terpolymer has not been previously known for such a complex architecture, but only for linear ABC triblock terpolymers of the PS-*b*-PI-*b*-PEO [41,42,43] and the PI-*b*-PS-*b*-P2VP sequences respectively [62]. Additionally, such a microdomain structure was also predicted [63] for linear terpolymers, when B is the matrix and A, C are the networks, by self-consistent mean-field theory when the interaction parameters between the blocks were: χ_A__B_~χ_BC_ < χ_AC_.

As reported in the literature [41,42,43] the structure corresponds to alternating Gyroid with two different component networks, which are also asymmetric. The TEM images for the [111] projection observed for the linear materials, in the aforementioned literature, are almost identical with those observed in our three-component miktoarm star. It should be mentioned that there is a distinct difference between the single gyroid and alternating gyroid exhibiting the I4_1_32 space group. The single gyroid has only one network whereas the alternating gyroid has two asymmetric networks. Of course, adopting such a structure by simply considering two networks is incorrect, since the architecture implies that the networks should touch (the single junction point overall is a strong structural constraint through the molecule’s architecture). One possibility might be that the twonetworks “inflate” until they touch each other in order for the topology to be correct. This assumption is verified by the bright-field TEM images in Figure 2, Figure 3 and Figure 4, where it is evident that the grey and dark areas (corresponding respectively to the PI and PB domains) are actually interconnected (which of course is a completely different observation when the DG or OBDD cubic structures are examined, where the two networks are interpenetrated but not interconnected).

Actually, the TEM images in both cases (linear from the Bates group [41,42,43] vs. nonlinear ABC terpolymer reported here) exhibited a three-phase contrast system, where the matrix was PS and the networks were the remaining two components. Examining the architecture of each material, the major difference is in the junction points: one in the miktoarm star terpolymer vs. two in the linear terpolymer. Despite this difference, it seems that in our case of the PS/PB-1,4/PI-3,4 system the molecular weights, volume fraction ratios and the interaction parameters are directing the structural behavior to be similar to other linear ABC type terpolymers. 

**Figure 6:**
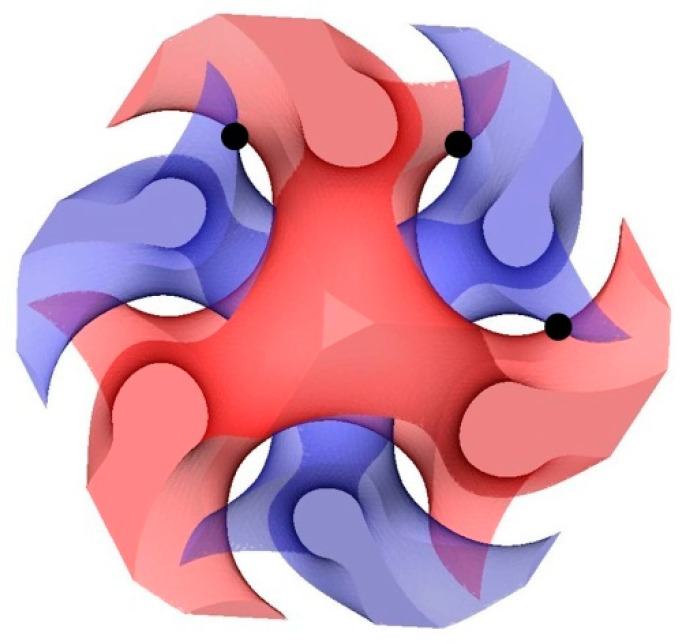
Schematic illustration of the two asymmetric networks of PI (blue color) and PB (red color) in PS matrix (white). The networks are inflated in order to reach the corresponding volume fractions of 0.23 and 0.31, respectively for the PI and PB segments. The black dots in the scheme indicate the possible junction point location of the ABC miktoarm terpolymer.

A possible schematic illustration of the adopted topology of the two polydiene networks in the PS matrix is given in Figure 6. It is evident that the location of the junction points (black dots in Figure 6) is not that straightforward since, according to the architecture limitations, all three blocks should intersect probably in lines and corner-type locations within the cubic overall domains.

The potential applications of such a system are exciting, since it could be used as a multifunctional sensor or as a multiselective catalyst for sequential or simultaneous chemical reactions of various kinds, as a nanoporous membrane with different porosities, etc. [64]. Similar systems based on the biological development of triply periodic, cubic, photonic crystals are considered promising in manufacturing convenient templates for the design of devices for photonic applications based on biomimicry or positive cast dielectric infiltration [65].

## 4. Conclusions

The synthesis of a 3-miktoarm star terpolymer with two polydiene components is reported. The increased 3,4-component in the PI microstructure leads to a three-microphase separation system (white: PS, grey: 3,4-PI and black: PB, by staining with OsO_4_), due to increased value of the χparameter between the polydiene segments. The morphological characterization suggests a cubic structure, with space group I4_1_32, reported previously in the literature only for linear ABC terpolymers. It seems that such an alternating gyroidstructure can be observed in both linear and nonlinear architectureterpolymers, modifying the fact that altering the architecture generally has a major impact on the observed microphase separated structures.

## Figures and Tables

**Table 1:** Molecular characteristics of the precursors and the final 3-miktoarm star terpolymer.

Sample	${\bar{M}}_{w}{\;}^{a}$ (g/mol)	${\bar{M}}_{n}{\;}^{b}$ (g/mol)	*Đ* ^c^	*f* (*w*/*w*) ^d^			(% *v*/*v*) ^e^			PI Microstructure (% wt) ^f^		
				**PI_3,4_**	**PS**	**PB**	**PI_3,4_**	**PS**	**PB**	**PI_3,4_**	**PI_1,2_**	**PI_1,4_**
Arm PI_3,4_	30.400	29.200	1.04							54	16	30
Arm PS	62.800	61.000	1.03							-	-	-
Arm PB	39.700	38.200	1.04								8 (PB_1,2_)	92 (PB_1,4_)
Miktoarm Star Terpolymer	139.800	130.700	1.07	0.22 (0.23)	0.48 (0.47)	0.30 (0.30)	0.23	0.46	0.31	54	16	30

^a^ LALLS in THF at 25 °C, ^b^ MO in toluene at 35 °C, ^c^ dispersity (Đ) calculated from SEC in ΤHF at 30 °C, ^d^ Mass fractions calculated from ^1^H-NMR in CDCl_3_ (in parentheses the values indicate the fractions calculated from the molecular weights using the equation: fi=(M¯n)i(M¯n)star), ^e^ Volume fractions calculated from^1^H-NMR mass fractions, by using the following densities of the chains: *d_PS_* = 1.06 g/mL, *d_PI_* = 0.903 g/mL and *d_PB_* = 0.9 g/mL), ^f^ from ^1^H-NMR of the PI_3,4_ arm of the miktoarm star in CDCl_3_.

**Table 2:** Calculation of Flory–Huggins interaction parameter χ.

Segments	χ ^a^	χ ^b^
	0.0940	0.0910
PS/PI_1,4_	0.0510	0.0450
PS/PB_1,2_	0.1140	-
PS/PI_3,4_	0.0940 ^c^	0.0910
PI_3,4_/PB_1,4_	0.0045 ^c^	-

^a^ By using Equation (1) of the manuscript. ^b^ From reference 40. ^c^ Due to the unknown density of the PI-3,4 block we used the density of the PI-1,4. The increased high 3,4-microstructure suggests a solubility parameter higher than that for cis-1,4-PI (90 wt %), therefore a lower value of χ_SI_ and χ_ΒΙ_.

**Table 3:** Permitted and experimentally observed reflections of the I4_1_32 cubic space group (according to International Tables of Crystallography). Comparison with the permitted reflections of $Pn\bar{3}m$(OBDD) and $Ia\bar{3}d$(DG).

*s*^2^ = *h*^2^ + *k*^2^ + *l*^2^	Permitted Reflections I4_1_32 (Single G)	Permitted Reflections $Pn\bar{3}m$(OBDD)	Permitted Reflections $Ia\bar{3}d$(DG)	Observed Reflections (SAXS)
2	[110]	[110]	-	[110]
3	-	[111]	-	-
4	-	[200]	-	-
6	[211]	[211]	[211]	[211]
8	[220]	[220]	[220]	[220]
9	-	[221]	-	-
10	[310]	[310]	-	-
11	-	[311]	-	-
12	[222]	[222]	-	-
14	[321]	[321]	[321]	[321]
16	[400]	[400]	[400]	-
17	-	[322]	-	-
18	[411] or [330]	[411] or [330]	-	-
19	-	[331]	-	-
20	[420]	[420]	-	-
21	-	[421]	-	-
22	[332]	[332]	[332]	-
24	[422]	[422]	[422]	-
26	[510] or [431]	[510]	[431]	[510]
